# Plasmonic Au@Ag Core–Shell Nanoisland Film for Photothermal Inactivation and Surface-Enhanced Raman Scattering Detection of Bacteria

**DOI:** 10.3390/nano14080695

**Published:** 2024-04-17

**Authors:** Sadang Husain, Chinmaya Mutalik, Sibidou Yougbaré, Chun-You Chen, Tsung-Rong Kuo

**Affiliations:** 1 International Ph.D. Program in Biomedical Engineering, College of Biomedical Engineering, Taipei Medical University, Taipei 11031, Taiwan; d845110001@tmu.edu.tw; 2 Department of Physics, Faculty of Mathematics and Natural Science, Lambung Mangkurat University, Banjarmasin 70124, Indonesia; 3 Graduate Institute of Nanomedicine and Medical Engineering, College of Biomedical Engineering, Taipei Medical University, Taipei 11031, Taiwan; cm121193@tmu.edu.tw; 4 Institut de Recherche en Sciences de La Santé/Direction Régionale du Centre Ouest (IRSS/DRCO), Nanoro BP 218, 11, Burkina Faso; ysibidou@gmail.com; 5 Artificial Intelligence Research and Development Center, Wan Fang Hospital, Taipei Medical University, Taipei 11696, Taiwan; 6 Department of Radiation Oncology, Wan Fang Hospital, Taipei Medical University, Taipei 11696, Taiwan; 7 Graduate Institute of Biomedical Informatics, College of Medical Science and Technology, Taipei Medical University, Taipei 11031, Taiwan; 8 Stanford Byers Center for Biodesign, Stanford University, Stanford, CA 94305, USA

**Keywords:** Au@Ag core–shell nanoisland films, surface-enhanced Raman scattering, bacteria, detection, photothermal therapy, theranostics

## Abstract

Plasmonic metal nanomaterials have been extensively investigated for their utilizations in biomedical sensing and treatment. In this study, plasmonic Au@Ag core–shell nanoisland films (Au@AgNIFs) were successfully grown onto a glass substrate using a seed-mediated growth procedure. The nanostructure of the Au@AgNIFs was confirmed through scanning electron microscopy (SEM), energy-dispersive X-ray spectroscopy (EDX), and atomic force microscopy (AFM). The UV-Vis spectra of the Au@AgNIFs exhibited a broad absorption in the visible range from 300 to 800 nm because of the surface plasmon absorption. Under simulated sunlight exposure, the temperature of optimal Au@AgNIF was increased to be 66.9 °C to meet the requirement for photothermal bacterial eradication. Furthermore, the Au@AgNIFs demonstrated a consistent photothermal effect during the cyclic on/off exposure to light. For photothermal therapy, the Au@AgNIFs revealed superior efficiency in the photothermal eradication of *Escherichia coli* (*E. coli*) and *Staphylococcus aureus* (*S. aureus*). With their unique nanoisland nanostructure, the Au@AgNIFs exhibited excellent growth efficiency of bacteria in comparison with that of the bare glass substrate. The Au@AgNIFs were also validated as a surface-enhanced Raman scattering (SERS) substrate to amplify the Raman signals of *E. coli* and *S. aureus*. By integrating photothermal therapy and SERS detection, the Au@AgNIFs were revealed to be a potential platform for bacterial theranostics.

## 1. Introduction

Plasmonic nanomaterials have gained widespread utilization in biomedical applications owing to their distinctive properties, such as a substantial surface area, facile surface modification, and unique surface plasmon resonance [1,2,3,4,5,6,7,8]. Among the array of plasmonic nanomaterials, silver-based nanomaterials with modifiable sizes and shapes have been extensively applied in therapeutic contexts, capitalizing on their structural and optical attributes [9,10,11]. Plasmonic silver-based nanomaterials have been demonstrated in phototherapy, particularly in cancer therapy, antimicrobial treatment, and wound healing, owing to their strong photothermal effects [12,13,14,15,16]. PEGylated bovine serum albumin-coated silver nanoparticles have revealed significant tumor accumulation, stability against degradation and hepatic clearance, leading to marked tumor growth inhibition upon local laser exposure [17]. Silver nanoparticle-conjugated graphene quantum dots have been demonstrated for the enhancement of bactericidal efficacy through blue light-activated photothermal therapy [18]. Hollow silver–gold alloy nanoparticles immobilized with a photosensitizer have been applied to combine photothermal therapy and photodynamic therapy to effectively eliminate bacteria and enhance wound healing by inducing mild hyperthermia and controlled singlet oxygen generation upon near-infrared laser activation [19]. Numerous studies have been undertaken to validate the photothermal therapy of plasmonic silver-based nanomaterials, renowned for their potent photothermal effects [20,21,22]. Nevertheless, the imperative need remains for the advancement of multifunctional silver-based nanomaterials in the realm of bacterial therapy to address the urgent challenge of treating bacterial infections.

Plasmonic silver nanomaterials have been also demonstrated for the detection applications because of their superior optical characteristic [23,24,25,26,27,28,29,30]. Based on strong surface plasmon resonance, silver nanomaterials have been extensively utilized for the detections of elements, molecules, proteins, and bacteria [31,32,33]. For example, silver nanocrystal Langmuir–Blodgett films with plasmonic properties have served as efficient matrix-free surfaces for applications in mass spectrometry to enhance signal intensity, reduce background noise, improve signal-to-noise ratio, and increase reproducibility [34]. Cuboctahedral silver nanocrystal films have been employed as surface-enhanced Raman scattering (SERS) substrates for quantitative analysis of the temporal and spatial variations in polyvinylpyrrolidone SERS signals by laser scanning confocal mapping [35]. Silver nanoparticles combined with mercaptoundecanoic acid have been demonstrated to have utility as surface plasmon resonance colorimetric sensors in the selective detection of micromolar levels of nickel ions in real time, even in the presence of various other metallic cations [36]. The achievements in plasmonic silver nanomaterials have shown significant promise in the biomedical realm for the detection of molecules, disease biomarkers, and heavy metals.

Recently, plasmonic metal nanoisland films have been engineered to enhance biomedical detection through superior plasmon-enhanced interactions such as fluorescence enhancement, photothermal effect, and SERS enhancement [37,38,39,40,41]. Moreover, with their excellent stability and high homogeneity, plasmonic metal nanoisland films have been proven to fulfill the practical requirements for biomedical therapy and detection. For example, for the detection of As(III), a fluorescence-enhanced assay has been developed via utilizing a carboxylic acid-functionalized silver nanoisland films (Au@AgNIFs) for the specific capture of As(III) ions and for the enhancement of fluorescent signals for the quantitative detection of As(III) [42]. By bridging microscopic mechanisms and macroscopic observations, Au@AgNIFs have exhibited a 488-fold fluorescence intensity enhancement of IR800-streptavidin within nanoisland valleys [43]. Plasmonic gold nanoisland films have served as SERS substrates for the significant enhancement of the Raman signal of *Escherichia coli* (*E. coli*) and exhibited remarkable efficiency for photothermal killing of *E. coli* under simulated sunlight exposure [44]. Although plasmonic gold nanoisland films have been validated for use in bacterial theranostics, there remains limited exploration of photothermal therapy and SERS detection based on Au@AgNIFs.

In this study, a straightforward seed-mediated growth technique was utilized for the deposition of Au@AgNIFs onto glass substrates. The structural and optical characteristics of the Au@AgNIFs were assessed using scanning electron microscopy (SEM), atomic force microscopy (AFM), and ultraviolet–visible (UV-Vis) spectroscopy. The photothermal effects of the Au@AgNIFs were explored under simulated sunlight exposure. Photothermal therapy experiments with the Au@AgNIFs were conducted on both the Gram-negative bacterium *E. coli* and Gram-positive bacterium *Staphylococcus aureus* (*S. aureus*). Additionally, the Au@AgNIFs were studied as a SERS substrate for enhancing Raman signals from both *E. coli* and *S. aureus*.

## 2. Materials and Methods

### 2.1. Materials

Silver nitrate (AgNO_3_), sodium borohydride (NaBH_4_), succinic anhydride 99% (C_4_H_4_O_3_), and N, N-diisopropylethylamine (DIPEA) were acquired from Acros Organics (Morris, NJ, USA). Ammonium hydroxide solution 30% (NH_4_OH) was purchased from Honeywell Fluka^TM^ (Loughborough, UK). Gold (III) chloride trihydrate (HAuCl_4_·3H_2_O) was purchased from Alfa Aesar (Haverhill, MA, USA). D-glucose, sodium hydroxide (NaOH), dimethylformamide (DMF, 99.5%), SYTOX green, and polysine substrates (2.5 cm × 7.5 cm × 0.1 cm) were purchased from Thermo Fisher Scientific (Waltham, MA, USA). Hoechst 33342 was purchased from Bio-genesis Technologies, Inc. (Taipei, Taiwan). Paraformaldehyde (PFA) was purchased from Sigma-Aldrich (St. Louis, MO, USA). Trypticase Soy Broth (TSB) Medium was purchased from Condalab (Madrid, Spain).

### 2.2. Synthesis of the Au@AgNIFs

Before the synthesis of the Au@AgNIFs, the glass substrates were sequentially washed using acetone, methanol, and deionized water under ultrasonication for 5 min. After that, to modify with the carboxylic group, 6 substrates were put into a Petri dish and then incubated in a solution containing succinic anhydride (0.45 g), DMF (25 mL) and DIPEA (0.606 mL) for 18 h in dark conditions. After incubation, the substrates were sequentially rinsed with ethanol and deionized water, followed by spin-drying. The substrates modified with the carboxylic group were stored at 4 °C. All glass containers were cleaned using an aqua regia solution. For the synthesis of the Au@AgNIFs, 6 substrates modified with the carboxylic group were immersed into 37.5 mL solution containing HAuCl_4_ (1 mM) and NH_4_OH (6%) at 10 °C for 20 min under shaking at 40 rpm to deposit Au^3+^ ions onto the substrate surface. Then, the substrates with Au^3+^ ions were washed using deionized water twice. After that, 6 substrates with Au^3+^ ions were moved to a new glass container containing 50 mL of deionized water and 50 mL of NaBH_4_ (2 mM) was added for 5 min to reduce Au^3+^ to Au seeds. Then, all of the substrates with Au seeds were rinsed again using deionized water and the back side of the substrates was cleaned using kimwipes after being immersed in deionized water and protected from light using aluminum foil. To grow the Au@AgNIFs, 100 mL of Tollens’ reagent solution (0.5 mM of AgNO_3_, 10 mM of NaOH, and 39.3 mM of NH_4_OH) was prepared. After adding 12.5 mM of D-glucose, the Tollens’ reagent solution was poured into the container containing substrates with Au seeds for various growth times at 30 °C. The substrates with the Au@AgNIFs were washed twice with deionized water and spin-dried. The Au@AgNIF substrates were stored at 4 °C for the following experiments.

### 2.3. Culture Bacteria on the Surfaces of the Au@AgNIFs for SEM Measurement

*S. aureus* and *E. coli* were used for bacterial theranostics. To culture bacteria, 6 g of TSB was dissolved into 200 mL of sterilized deionized water. Then, the TSB medium was sterilized again in an autoclave chamber for 90 min. Next, 20 µL of the bacterial solution was cultured in 3 mL of TSB medium in a culture tube under shaking at 170 rpm and 37 °C overnight. Afterwards, the Au@AgNIFs with a size of 1 × 1 cm^2^ were put into a small Petri dish, and 3 mL of bacterial solution was added with a value of optical density at 600 nm (OD600) at 0.1. The Petri dish was placed in an incubator at 37 °C for 30 min. Then, the substrate was washed using deionized water 3 times and 4% PFA sequentially added for 30 min. PFA was used as a fixation agent for bacteria for SEM measurement. Eventually, the substrate was washed with 70%, 80%, 90%, 95%, and 100% ethanol. Finally, the bacteria on Au@AgNIFs were examined by SEM.

### 2.4. Characterizations of the Au@AgNIFs

A UV-Vis spectrophotometer (JASCO V-770, JASCO Corporation, Tokyo, Japan) was used to confirm the absorption of the Au@AgNIFs. The UV-Vis spectrophotometer was operated at the wavelength of 300–800 nm, bandwidth of 1 nm, and scan speed of 1000 nm/min. SEM (SU3500 Hitachi Ltd., Hitachi, Japan) was applied to examine the surface morphology of the Au@AgNIFs. For the observation, 10 kV and 23,000-fold magnification was used in the instrument. AFM (FSM-Nanoview1000, UTEK MATERIAL, Taipei City, Taiwan) was carried out to observe the 3D structure of Au@AgNIFs. The Au@AgNIF specimens, each measuring 1 × 1 cm, were mounted onto the holder and subjected to testing at a frequency of 150 kHz, utilizing a scanning size of 2000 μm. Subsequently, the results were analyzed using WSxM v5.0 software to visualize the morphology.

### 2.5. Photothermal Assessment

The Au@AgNIFs were irradiated by stimulated AM1.5 sunlight with various power densities, including 50, 100, 150, 200, 250, and 300 mWcm^−2^. The temperature of the Au@AgNIFs was checked every min by a thermal camera (FLIR TG267) (Teledyne FLIR, Wilsonville, CA, USA). The measurement was carried out for 20 min. Besides that, the other treatment used was on and off alternating stimulation by AM1.5 sunlight. The sunlight was turned on for 10 min and turned off for 10 min. The cycle was performed 3 times.

### 2.6. Bacterial Growth under Different Temperatures

Bacterial suspensions were cultured at 37, 45, 50, 55, and 60 °C in culture tubes shaken at 170 rpm. A cell density meter Ultrospec 10 (Cold Spring Biotech Corp., New Taipei City, Taiwan) was used to monitor bacterial density at 30 min intervals for a total of 360 min in order to assess the impact of temperature on bacterial growth.

### 2.7. Bacterial Inhibition by the Au@AgNIFs under Light Exposure

The glass substrate containing the Au@AgNIFs was initially cultivated with bacteria (*S. aureus* and *E. coli*) suspensions with an OD600 value of 0.1 in culture dishes at 37 °C for 2 h. To examine bacterial inhibition under light exposure, simulated AM1.5 sunlight (300 mW/cm^2^) was then used to irradiate the bacteria-containing Au@AgNIFs for 10 min. The control experiments were executed without light exposure. Herein, Hoechst 33342 and SYTOX green were applied to measure the total number and number of dead bacteria, respectively. Hoechst 33342 and SYTOX green are fluorescent dyes commonly utilized in microbiology for staining bacteria and cells, aiding in their visualization and quantification [45,46]. Hoechst 33342, classified as a DNA stain, has the ability to permeate into cell membranes and specifically bind to the minor groove of double-stranded DNA. When exposed to ultraviolet light, Hoechst 33342 emits blue fluorescence, enabling observation under a fluorescence microscope [47]. The quantification of fluorescently labeled bacteria allows for the estimation of the bacterial population present on a surface [48]. For another DNA stain, although SYTOX green cannot penetrate intact cell membranes, it can enter cells with compromised membranes, such as those of dead or dying cells. Once inside the cell, SYTOX green binds to DNA and emits green fluorescence when exposed to ultraviolet light or blue light [49]. Staining bacteria with SYTOX green and observing the green fluorescence under a microscope allows for the differentiation between live and dead bacteria on a surface [50]. Bacteria mortality was assessed by fluorescence imaging after exposure to light using SYTOX green staining (5 µM) for 30 min. Afterward, SYTOX green was removed from the dish and washed using Hanks’ Balanced Salt Solution (HBSS) for 10 min. Repetition of washing was performed 3 times. Live/dead cells of bacteria were checked by fluorescence imaging with marked Hoechst 33342 staining (15 µg/mL) for 30 min in a culture dish. After that, staining was removed from the culture dish and washed using phosphate-buffered saline (PBS) for 10 min, which was carried out 3 times. Dead bacteria were stained with SYTOX green (resulting in a green pseudocolor) while entire bacteria were stained with Hoechst 33342 before being viewed using a fluorescent microscope (Leica DMi8) (Wetzlar, Germany) (producing a blue pseudocolor).

### 2.8. Raman Spectroscopy and Measurement

To detect bacteria, laser confocal Raman spectroscopy (UniDRON, Taipei City, Taiwan), was employed following the outlined procedure. A bacterial solution (0.1 mL) with an OD600 value of 0.5 was mixed with 0.9 mL of PBS. The resultant mixture underwent centrifugation at 2000 rpm for 2 min, causing the bacteria to precipitate onto the surfaces of both the Au@AgNIF and glass substrate. Subsequently, after drying in the incubator for 15 min, the surfaces of Au@AgNIFs and glass substrates containing bacteria were examined using laser confocal Raman spectroscopy with a 632 nm laser, 50-fold microscope magnification, and 5% intensity.

## 3. Results

### 3.1. Structural and Optical Properties of the Au@AgNIFs

**Figure 1:**
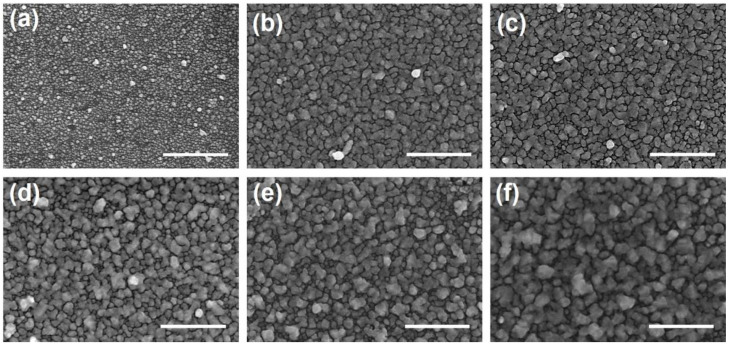
SEM images of the Au@AgNIFs with the growth times of (**a**) 3, (**b**) 4, (**c**) 5, (**d**) 6, (**e**) 7, and (**f**) 8 min. All scale bars are 1 μm.

The Au@AgNIFs were synthesized by depositing Au^3+^ ions followed by Au seeds and finally the growth of the Ag shell to form Au@Ag core–shell nanoislands. Photographic images of Au@AgNIFs during synthesis are shown in Figure S1 in the Supporting Information. The morphology of the Au@AgNIFs was first characterized by SEM. As shown in the SEM images of Figure 1, the growth times of the Au@AgNIFs are 3, 4, 5, 6, 7, and 8 min in Figure 1a, Figure 1b, Figure 1c, Figure 1d, Figure 1e and Figure 1f, respectively. In Figure 1, the nanoislands are distributed on the entire substrate. Furthermore, with the increase in growth time, the separated nanoislands (Figure 1a) gradually merged into large and convoluted nanoislands. As the size of the nanoislands increased, the gap distance of the Au@AgNIFs significantly decreased. For SERS measurements, the gap distance of the Au@AgNIFs is an important factor. The average gap distances of the Au@AgNIFs were calculated to be 38.9, 27.3, 21.6, 15.7, 12.6, and 10.2 nm for the growth times of 3, 4, 5, 6, 7, and 8 min, respectively. The best SERS enhancement was demonstrated with an average gap distance of around 15 nm [43]. Therefore, the Au@AgNIFs with the growth time of 6 min (average gap distance was 15.7 nm) were selected for the following experiments. To further confirm the composition of nanoislands, the EDX was applied to analyze the Au@AgNIFs with the growth time of 6 min as shown in Figure S2. The elemental analysis indicated that the Au@AgNIFs comprised silver and gold, with atomic% of 40.59 and 59.41%, respectively. The gold came from the gold seeds and the silver came from the nanoislands. Therefore, the EDX analysis of the Au@AgNIFs verified the successful preparation of silver nanoislands on the glass substrate.

**Figure 2:**
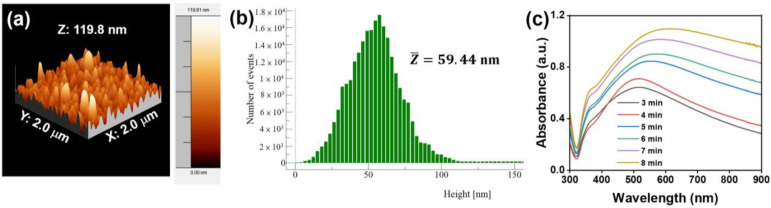
(**a**) AFM image of the Au@AgNIFs with the growth time of 6 min. (**b**) Histogram of height distribution of silver nanoislands in Au@AgNIFs with the growth time of 6 min. The average height was calculated to be 59.44 nm. (**c**) UV-Vis absorption spectra of the Au@AgNIFs with different growth times, including 3, 4, 5, 6, 7, and 8 min.

To further evaluate the surface morphology of the Au@AgNIFs, AFM was executed to measure the surface roughness. As shown in the AFM image of Figure 2a, the Au@AgNIFs with the growth time of 6 min revealed the shape of the silver nanoislands on the entire substrate. The highest silver nanoisland was measured to be 119.8 nm. To analyze the average height, the height distributions of the silver nanoislands were measured according to the AFM image of Figure 2a. The histogram of the height distribution of silver nanoislands was shown in Figure 2b. According to the histogram of Figure 2b, the average height was measured to be 59.44 nm. Moreover, the optical property of the Au@AgNIFs was carried out using UV-Vis spectroscopy. As shown in Figure 2c, the Au@AgNIFs with various growth times from 3 to 8 min exhibited a broad absorption in the visible range from 300 to 800 nm because of the surface plasmon absorption. With the increase in the growth time, the absorption spectra of Au@AgNIFs revealed red shift and an increase in absorption intensity due to the increase in the size of the silver nanoislands, corresponding to the SEM images of Figure 1. Overall, based on the results of the structural and optical properties, the plasmonic Au@AgNIFs have shown a promising potential as a SERS substrate for bacterial theranostics.

### 3.2. Photothermal Therapy of the Au@AgNIFs

**Figure 3:**
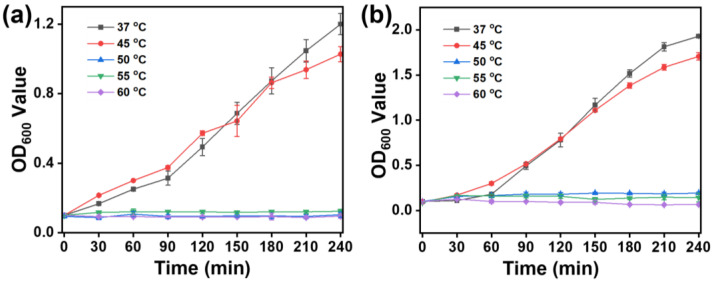
Bacterial growth curves were recorded at various culture temperatures. (**a**) *E. coli* and (**b**) *S. aureus*. Bacterial OD600 values were measured at 30 min intervals.

In order to investigate the impact of temperature on photothermal therapy, bacteria of *E. coli* and *S. aureus* were, respectively, subjected to various temperature conditions, including 37, 45, 50, 55, and 60 °C. As shown in Figure 3a, under the standard culture temperature of 37 °C, *E. coli* exhibited robust exponential growth. After a 360 min culture period, the OD600 reached a value of 1.38, indicating a healthy proliferation of *E. coli*. However, when the culture temperature was increased to 45 °C, a minor growth inhibition of *E. coli* was observed. After the same 360 min culture duration, the OD600 value of *E. coli* culture was measured at 1.19. Most notably, at temperatures of 50, 55, and 60 °C in the culture, *E. coli* growth curves revealed a substantial absence of growth. After the 360 min culture period under these elevated temperatures, the OD600 values of *E. coli* were only 0.10, 0.18, and 0.14, respectively. Furthermore, *S. aureus* was also exposed to varying temperatures (37, 45, 50, 55, and 60 °C) to assess their impact on growth. As shown in Figure 3b, at the standard 37 °C culture temperature, *S. aureus* exhibited robust exponential growth, with an OD600 value reaching >2.0 after 360 min. When cultured at 45 °C, there was a slight growth inhibition, resulting in an OD600 value of 2.0 at the same culture time of 360 min. The bacteria of *S. aureus* failed to demonstrate significant growth at 50, 55, and 60 °C, with OD600 values of 0.19, 0.16, and 0.11, respectively. These results clearly indicated that the bacteria of *E. coli* and *S. aureus* used in this study cannot survive temperatures exceeding 50 °C.

**Figure 4:**
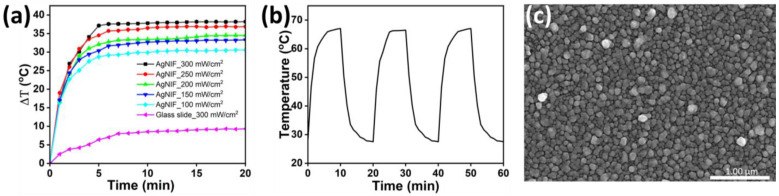
(**a**) Temperature measurement of the Au@AgNIFs and glass slide for checking photothermal performance under simulated sunlight exposure. (**b**) Photothermal response of the Au@AgNIFs under on/off cycles of simulated sunlight exposure at a power density of 300 mW/cm^−2^. (**c**) SEM image of the Au@AgNIFs after light exposure.

To demonstrate the photothermal effect, the Au@AgNIFs with the growth times of 3, 4, 5, 6, 7, and 8 min were exposed to simulated sunlight (300 mW/cm). As shown in the Supporting Information of Figure S3, the Au@AgNIFs with a growth time of 6 min exhibited the best photothermal performance. To further determine photothermal response, the Au@AgNIFs with the growth time of 6 min were exposed to simulated sunlight at power densities of 150, 200, 250, and 300 mW/cm. A thermal camera (FLIR TG267, Teledyne FLIR, Wilsonville, CA, USA) was used to measure the temperature of the Au@AgNIFs under light exposure. As a control, the glass slide was exposed to simulated sunlight at a constant 300 mW/cm^2^. Figure 4a illustrates that the temperature of the glass slide did not exhibit a substantial increase (temperature difference less than 10 °C) during light exposure. In contrast, when the Au@AgNIFs were exposed to light at power densities of 150, 200, 250, and 300 mW/cm^2^ for 2 min, their temperature increased to >50 °C (temperature difference >20 °C), meeting the criteria for photothermal bacteria eradication. To illustrate the reversibility of the photothermal impact, the Au@AgNIFs underwent cycles of light exposure at 300 mW/cm^2^ for 10 min, followed by 10 min without light, repeated three times. Figure 4b illustrates the temperature changes during these cycles. The results showed that, after each light exposure period, the temperature of the Au@AgNIFs reached 66.9 °C. Importantly, the photothermal effect of the Au@AgNIFs exhibited a consistent response to the on/off light exposure cycling. Additionally, SEM analysis revealed that the silver nanoislands of the Au@AgNIFs remained largely unchanged after the on/off light exposure, as depicted in Figure 4c. In summary, the reusable Au@AgNIFs demonstrated a reliable and reversible photothermal effect, showcasing their potential for applications in photothermal bacteria eradication.

**Figure 5:**
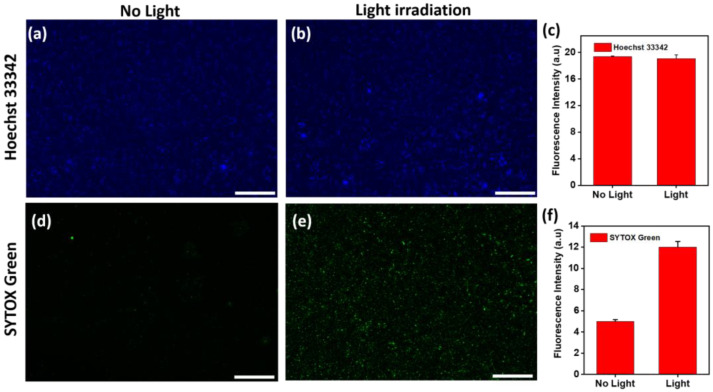
Fluorescent images depicting Hoechst 33342 staining of Au@AgNIFs in the presence of *E. coli* (**a**) without light exposure and (**b**) with light exposure. Corresponding fluorescence intensities are represented in (**c**). Furthermore, SYTOX green fluorescence images for Au@AgNIF cultured with *E. coli* are provided (**d**) without light exposure and (**e**) with light exposure. The associated fluorescence intensities are shown in (**f**). Scale bars for all images are 100 µm.

To assess the effectiveness of photothermal therapy, the Au@AgNIFs cultured with *E. coli* and *S. aureus* were, respectively, exposed with and without simulated sunlight to assess the efficacy of photothermal treatment. To measure fluorescence, the parameters of magnification (10×), exposure time (1 ms), objective (10×/0.25 DRY), gain (7.6), and acquisition time (87 ms) were applied to fluorescence microscopy. As shown in Figure 5a,b, the blue fluorescence of Hoechst 33342 indicated no significant difference in the total number of *E. coli* cultured on the Au@AgNIFs, whether exposed to light or not for 10 min. In Figure 5c, the fluorescence intensities were calculated to be 19.37 ± 0.12 (no light) and 19.08 ± 0.55 (light exposure), confirming the similarity in the total number of *E. coli*. To assess the viability of *E. coli*, SYTOX green fluorescence was employed. Figure 5d,e demonstrate that light exposure led to higher SYTOX green fluorescence intensity compared to the condition without light. In Figure 5f, the fluorescence intensities were also calculated to be 4.99 ± 0.16 and 12.01 ± 0.53 for without and with light exposure, respectively. With light exposure, the SYTOX green fluorescence intensity of *E. coli* cultured with the Au@AgNIFs was 2.4 times higher than without light exposure. This increase was attributed to the plasmonic Au@AgNIFs absorbing light and converting it into heat through a photothermal effect, effectively killing *E. coli*.

**Figure 6:**
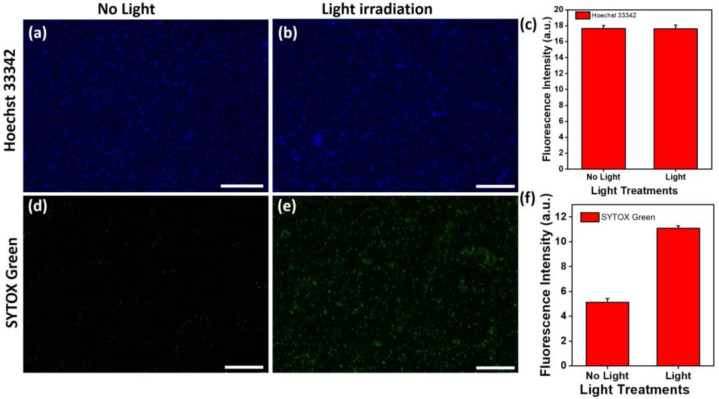
Fluorescent images illustrating Hoechst 33342 staining on Au@AgNIFs cultured with *S. aureus* (**a**) without light exposure and (**b**) with light exposure. Corresponding fluorescence intensities are depicted in (**c**). Additionally, SYTOX green fluorescence images of Au@AgNIF cultured with *S. aureus* are presented (**d**) without light exposure and (**e**) with light exposure. The associated fluorescence intensities are shown in (**f**). Scale bars for all images are 100 µm.

Moreover, with culturing on the Au@AgNIFs, *S. aureus* exhibited similar phenomena to *E. coli* both in the presence and absence of light exposure. As shown in the fluorescence images of Figure 6a,b, there was no significant difference in the total numbers of *S. aureus* cultured on the Au@AgNIFs with and without light exposure, as indicated by the blue fluorescence of Hoechst 33342. The fluorescence intensities with and without light exposure were calculated to be 17.60 ± 0.49 and 17.67 ± 0.36, respectively, as shown in Figure 6c. To further assess the viability, the SYTOX green fluorescence images of *S. aureus* on the Au@AgNIFs were also examined with and without light exposure as shown in Figure 6d,e. In Figure 6f, the fluorescence intensities were, respectively, measured to be 4.80 ± 1.66 and 11.07 ± 4.50 for Figure 6d,e. In the presence of light exposure, the SYTOX green fluorescence intensity of *S. aureus* cultured with the Au@AgNIFs was 2.3 times greater than that without light exposure. Overall, upon light exposure, the plasmonic Au@AgNIFs can absorb light and then convert it to heat via a photothermal effect, leading to the photothermal inactivation of bacteria. The results indicate that plasmonic Au@AgNIFs have great potential for antibacterial application via photothermal therapy.

### 3.3. Au@AgNIFs as a SERS Substrate for Bacterial Detection

**Figure 7:**
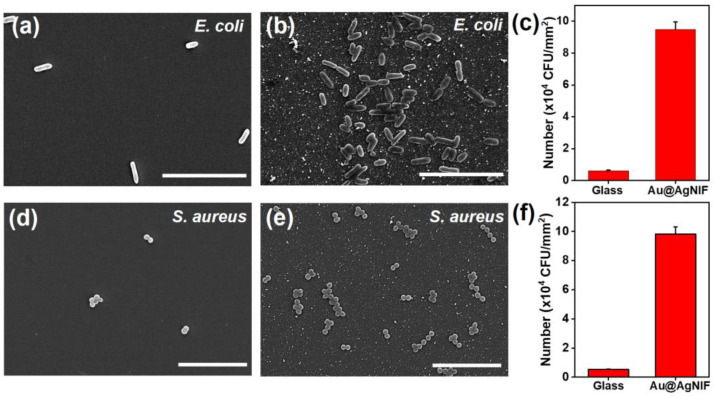
SEM images of *E. coli* (**a**) on the bare glass substrate and (**b**) on the Au@AgNIFs. (**c**) Statistical calculation of *E. coli* from (**a**,**b**). SEM images of *S. aureus* (**d**) on the bare glass substrate and (**e**) on the Au@AgNIFs. (**f**) Statistical calculation of *S. aureus* from (**d**,**e**). All scale bars were 10 µm.

Bacteria were cultured on both bare glass substrate and the Au@AgNIFs to assess their potential as SERS substrates for bacterial detection. As shown in Figure 7a, the SEM image revealed scattered *E. coli* on the bare glass surface. In the SEM image of Figure 7b, the Au@AgNIF surface had a significantly larger number of *E. coli* compared to that of the bare glass substrate. For quantitative analysis of *E. coli* growth efficiency, the quantities of *E. coli* on the bare glass substrate and Au@AgNIFs were independently calculated based on their SEM images as shown in Figure 7c. The number of *E. coli* colony-forming units (CFUs)/mm^2^ was calculated to be 6163 on the bare glass substrate and 94,898 on Au@AgNIFs. In comparison to the bare glass substrate, the Au@AgNIFs exhibited a 15.4-fold higher efficiency in growing *E. coli*. Furthermore, in Figure 7d,e, the SEM images indicated *S. aureus* on the bare glass substrate and on the Au@AgNIFs, respectively. The interaction between the Au@AgNIFs and *S. aureus* was significantly more pronounced than that observed with the bare glass substrate. The numbers of *S. aureus* on the bare glass substrate and the Au@AgNIF were calculated to be 5353 and 98,176 CFUs/mm^2^, respectively, demonstrating that Au@AgNIFs were 18.3 times more effective in interacting with *S. aureus*. The results suggest that the surface topography of the nanoislands on the Au@AgNIFs enhanced the growth of both *E. coli* and *S. aureus*. Overall, the Au@AgNIFs hold promise as a substrate with high bacterial growth efficiency, potentially paving the way for their application in bacterial SERS detection.

**Figure 8:**
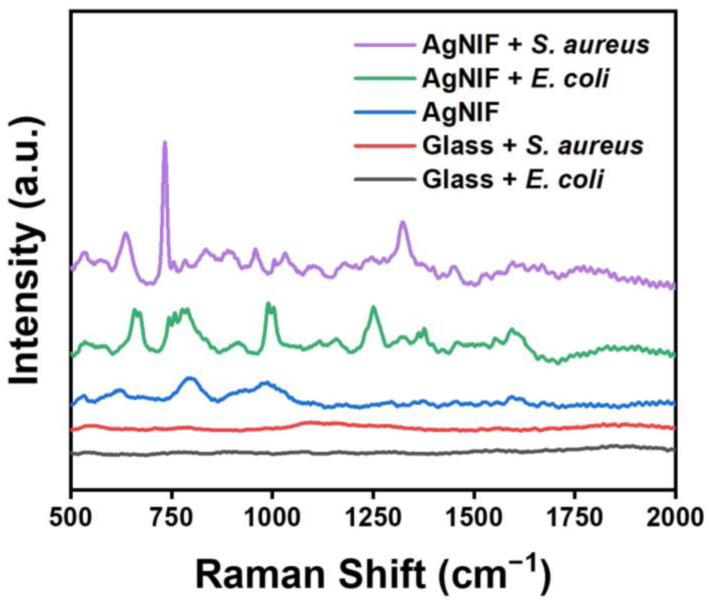
Raman spectra of the glass substrate cultured with *E. coli* (black line), glass substrate cultured with *S. aureus* (red line), Au@AgNIFs (blue line), Au@AgNIFs cultured with *E. coli* (green line), and Au@AgNIFs cultured with *S. aureus* (purple line).

To identify bacteria, Raman spectroscopy was employed to analyze the glass substrate and Au@AgNIFs with *E. coli* and *S. aureus*, respectively. As shown in Figure 8, the glass substrate cultured with *E. coli* or *S. aureus* did not exhibit a discernible Raman signal. In the case of Au@AgNIFs without bacteria, two Raman signals at 794 and 986 cm^−1^ were observed, attributed to the characteristic peak of carboxyl groups on the Au@AgNIF surface. The carboxyl groups were modified on the glass substrate for gold seed growth. After culture of *E. coli* on the Au@AgNIFs, characteristic Raman signals of *E. coli* emerged at 634, 733, 834, 957, 1002, 1032, 1247, 1322, 1448, and 1593 cm^−1^ (Table 1) [51,52,53]. Following the cultivation of *S. aureus* on the Au@AgNIFs, distinctive Raman signals associated with *S. aureus* were identified at 742, 990, 1159, 1250, 1322, 1452, and 1592 cm^−1^ (Table 1). The Raman measurements demonstrated the potential of plasmonic Au@AgNIFs as a promising SERS substrate for amplifying the Raman signals of bacteria.

## 4. Conclusions

A glass substrate was employed for the deposition of large-scale Au@AgNIFs using a straightforward seed-mediated growth technique. The structural characterization of the silver nanoislands and plasmonic absorption of Au@AgNIFs were, respectively, elucidated through SEM, AFM, and UV-Vis spectroscopy. Concerning the photothermal effect, exposure to simulated sunlight at a power density of 300 mW/cm^2^ for 10 min elevated the temperature of Au@AgNIFs to 66.9 °C. The Au@AgNIFs exhibited excellent reversibility of the photothermal effect and responsive behavior to on/off light exposure. In terms of photothermal therapy, the Au@AgNIFs demonstrated higher efficiency in the photothermal eradication of both *E. coli* and *S. aureus* compared to the bare glass substrate. Furthermore, the growth efficiencies of *E. coli* and *S. aureus* on the Au@AgNIFs exceeded those on the plain glass substrate, attributed to the distinctive surface topography of the silver nanoislands. In SERS applications, the Raman signals of *E. coli* and *S. aureus* cultured on the plasmonic Au@AgNIF surface exhibited noteworthy enhancement in comparison to signals obtained on the bare glass substrate. Based on the substrate of Au@AgNIFs, SERS detection can provide advantages such as high sensitivity and specificity, resistance to photobleaching, and compatibility with label-free detection, making it a valuable tool for detecting bacteria in various applications, including biomedical diagnostics and environmental monitoring. This study highlights the potential of plasmonic Au@AgNIFs as a versatile SERS platform for bacterial theranostics.

## Figures and Tables

**Table 1:** The Raman peaks of *E. coli* and *S. aureus* on the Au@AgNIFs.

Peak Position (cm^−1^)		Peak Assignment
*E. coli*	*S. aureus*	
634		C–C twisting
733	742	Adenine, glycosidic ring mode
834		Tyrosine
957		Bending (C=C) or tyrosine
1002	990	Phenylalanine or glucose
1032		C-H deformation
1168	1160	12-Methylpalmitic acid or aceto acetate
1247		Amide III
	1250	Cytosine, adenine
1322	1322	Stretching (NH_2_) adenine, polyadenine, DNA
1448	1452	CH_2_ deformation mode of proteins
1593	1592	CN stretching of Amide II

## Data Availability

All data are contained within the article.

## Supplementary Materials

The following supporting information can be downloaded at: https://www.mdpi.com/article/10.3390/nano14080695/s1, Figure S1. Photographic images of Au@AgNIFs during synthesis (a) deposition of Au^3+^ ions, (b) growth of Au seeds, and (c) growth of Ag shells. Figure S2. EDX analysis of Au@AgNIF (a) SEM image, (b) Ag mapping, (c) Au mapping, (d) EDX spectrum, and (e) elemental composition. Figure S3. Photothermal performances of Au@AgNIFs with the growth times of 3, 4, 5, 6, 7, and 8 min, respectively.
