# Supplementary material for: Plasmonic Au@Ag Core–Shell Nanoisland Film for Photothermal Inactivation and Surface-Enhanced Raman Scattering Detection of Bacteria
Source: Nanomaterials (Basel). 2024 Apr 17;14(8):695. doi: 10.3390/nano14080695 (PMC11053632; doi:10.3390/nano14080695)
Supplement: Supplementary file 1 [file nanomaterials-14-00695-s001.zip › nanomaterials-2956130-supplementary.pdf]

nanomaterials

Supporting Materials

Plasmonic Au@Ag Core-Shell Nanoisland Film for Photothermal Inactivation and SERS Detection of Bacteria

Sadang Husain [1,2], Chinmaya Mutalik [3], Sibidou Yougbaré [4], Chun-You Chen [5,6,7,*] and Tsung-Rong Kuo [1,3,8,*]

[1] International Ph.D. Program in Biomedical Engineering, College of Biomedical Engineering, Taipei Medical University, Taipei 11031, Taiwan; d845110001@tmu.edu.tw
[2] Department of Physics, Faculty of Mathematics and Natural Science, Lambung Mangkurat University, Banjarmasin 70124, Indonesia
[3] Graduate Institute of Nanomedicine and Medical Engineering, College of Biomedical Engineering, Taipei Medical University, Taipei 11031, Taiwan; cm121193@tmu.edu.tw
[4] Institut de Recherche en Sciences de La Santé/Direction Régionale du Centre Ouest (IRSS/DRCO), Nanoro BP 218, 11, Burkina Faso; ysibidou@gmail.com
[5] Artificial Intelligence Research and Development Center, Wan Fang Hospital, Taipei Medical University, Taipei 11696, Taiwan
[6] Department of Radiation Oncology, Wan Fang Hospital, Taipei Medical University, Taipei 11696, Taiwan
[7] Graduate Institute of Biomedical Informatics, College of Medical Science and Technology, Taipei Medical University, Taipei 11031, Taiwan
[8] Stanford Byers Center for Biodesign, Stanford University, Stanford, CA 94305, USA
* Correspondence: Correspondence: wfrtmouse@gmail.com (C.-Y.H.); trkuo@tmu.edu.tw (T.-R.K.)

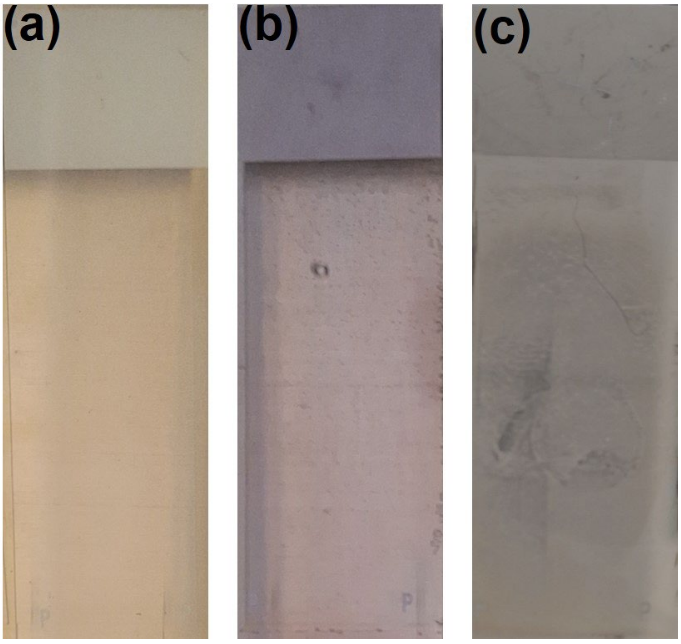

Figure S1. Photographic images of Au@AgNIFs during synthesis (a) deposition of Au^{3+} ions, (b) growth of Au seeds, and (c) growth of Ag shells.

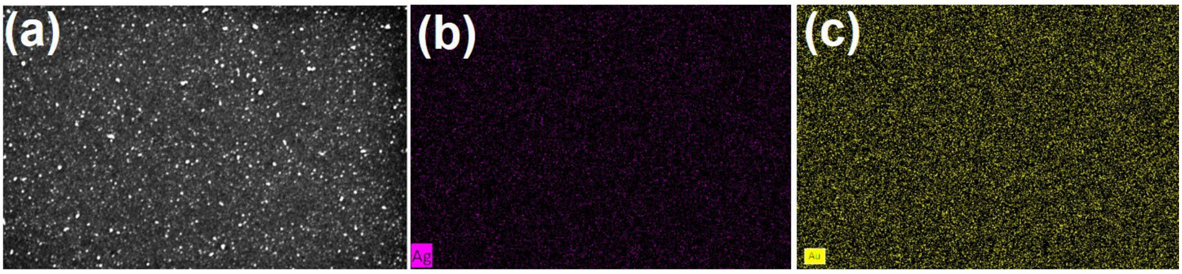

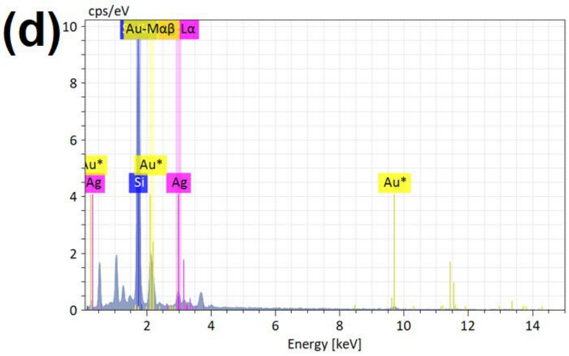

(e)

Element	Mass (%)	Mass Norm. (%)	Atom (%)
Gold	50.74	72.77	59.41
Silver	18.99	27.23	40.59
**Sum**	**69.73**	**100.00**	**100.00**

Figure S2. EDX analysis of Au@AgNIF (a) SEM image, (b) Ag mapping, (c) Au mapping, (d) EDX spectrum, and (e) elemental composition.

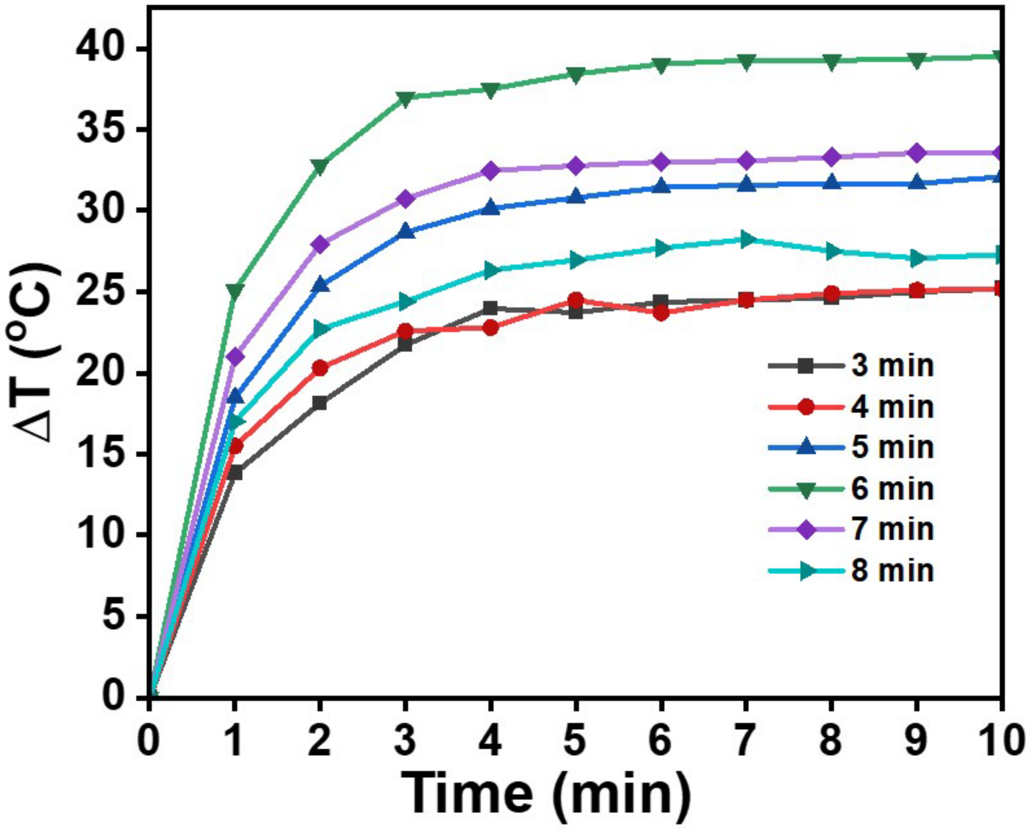

Figure S3. Photothermal performances of Au@AgNIFs with the growth times of 3, 4, 5, 6, 7, and 8 min, respectively.
